# A DLSTM-Network-Based Approach for Mechanical Remaining Useful Life Prediction

**DOI:** 10.3390/s22155680

**Published:** 2022-07-29

**Authors:** Yan Liu, Zhenzhen Liu, Hongfu Zuo, Heng Jiang, Pengtao Li, Xin Li

**Affiliations:** 1 Civil Aviation Key Laboratory of Aircraft Health Monitoring and Intelligent Maintenance, College of Civil Aviation, Nanjing University of Aeronautics and Astronautics, Nanjing 210016, China; jiayinly@nuaa.edu.cn (Y.L.); rms@nuaa.edu.cn (H.Z.); jiangheng@nuaa.edu.cn (H.J.); lipengtaomn@163.com (P.L.); lixin1990@nuaa.edu.cn (X.L.); 2 School of Automotive & Rail Transit, Nanjing Institute of Technology, Nanjing 211167, China

**Keywords:** remaining useful life prediction, data mining, kernel principal component analysis, maximal information coefficient, long short-term memory, deep learning

## Abstract

Remaining useful life prediction is one of the essential processes for machine system prognostics and health management. Although there are many new approaches based on deep learning for remaining useful life prediction emerging in recent years, these methods still have the following weaknesses: (1) The correlation between the information collected by each sensor and the remaining useful life of the machinery is not sufficiently considered. (2) The accuracy of deep learning algorithms for remaining useful life prediction is low due to the high noise, over-dimensionality, and non-linear signals generated during the operation of complex systems. To overcome the above weaknesses, a general deep long short memory network-based approach for mechanical remaining useful life prediction is proposed in this paper. Firstly, a two-step maximum information coefficient method was built to calculate the correlation between the sensor data and the remaining useful life. Secondly, the kernel principal component analysis with a simple moving average method was designed to eliminate noise, reduce dimensionality, and extract nonlinear features. Finally, a deep long short memory network-based deep learning method is presented to predict remaining useful life. The efficiency of the proposed method for remaining useful life prediction of a nonlinear degradation process is demonstrated by a test case of NASA’s commercial modular aero-propulsion system simulation data. The experimental results also show that the proposed method has better prediction accuracy than other state-of-the-art methods.

## 1. Introduction

With the rapid development of intelligent manufacturing and industrial internet of things technology, the mechanical equipment condition monitoring system collects a huge amount of data. These condition monitoring systems usually contain various sensors that provide a wealth of monitoring information offering new opportunities for predicting the remaining useful life (RUL) of machinery. At the same time, however, the data collected by these sensors is explosive and non-linear compared to traditional industrial monitoring data, making it challenging to use these data better [1]. Due to the complexity of current mechanical systems, it is exceedingly challenging to build an accurate mathematical or physical prognostics model based on fundamental principles of failure processes [2].

Data-driven RUL prediction is generally based on the following six processes: data acquisition, feature selection, data processing, feature extraction, degenerate behavior learning, and RUL prediction [3]. Firstly, the sensors installed on the machinery collect different monitoring data, such as vibration, pressure, temperature, and sound. Secondly, before analyzing the data, the data type and scale need to be clarified, and the data need to be pre-processed, such as with standardization after having a preliminary understanding of the data to support subsequent analysis and modeling of the data. In the third step, to map the machine’s health state, representative features need to be selected using signal processing techniques. However, these initially selected features may not be responsive to the deterioration of the system or provide any information that is valuable for RUL prediction. Therefore, there is also a need to use feature extraction methods in the aforementioned features to improve the sensitivity to the degradation state of the device. Muhammad Mohsin Khan et al. [4] utilized the obtained vibration signals from slurry pumps for generating degradation trends. Fernando Sánchez Lasheras et al. [5] combined the multivariate adaptive regression splines technique with the principal component analysis (PCA), dendrograms, and classification and regression trees to extract elements from sensor signals and train a hybrid model. Aleksandar Brkovic et al. [6] extracted the standard deviation and the logarithmic energy entropy as the representative features from interest sub-bands and reduced feature space dimension into two by scattering matrices. After that, these selected and extracted features were then fed into machine learning models to learn the degenerate behavior of the machine, such as Gaussian process regression [7], SVM [8], and artificial neural networks [9]. The RUL was then computed using these learnt models.

Deep learning [10] is gaining popularity for RUL prediction of machinery as a result of its potent data characterization capacity in data-driven RUL prediction. Deep learning is a subset of machine learning that employs multilayer artificial neural networks to provide cutting-edge accuracy in different classification or regression problems. Deep learning techniques, such as deep belief network (DBN) [11], recurrent neural network (RNN) [12], convolutional neural network (CNN) [13], and long short-term memory (LSTM) network [14], can automatically learn multiple levels of representation from raw data without the need for domain-specific expertise. Deep learning has been a considerable success in a variety of disciplines, including face recognition [15], speech recognition [16], and natural language processing [17], as a result of its full representational learning potential. Several deep learning-based studies have been conducted for the prediction of RUL in mechanical systems. Chen et al. [18] used a neural network with gated recurrent units to forecast the mechanical RUL of a nonlinear deterioration process. Zhang et al. [19] studied a new architecture of LSTM-based networks for discovering basic patterns in time series to track the degradation of the system and predict RUL. Wang et al. [20] proposed a deep separable conversation network, and this new deep prediction network can be used for mechanical RUL prediction. Kang et al. [21] developed an algorithm for automatically predicting mechanical failures in continuous production lines on the basis of a machine learning approach. Ji et al. [22] adopted the PCA–BLSTM model for the RUL prediction of an airplane engine.

Although deep learning has yielded fruitful results on mechanical RUL prediction, the existence of prediction methods still has the following limitations. (1) Correlations between data from different sensors and mechanical degradation information in deep learning algorithms are not explicitly considered in feature selection. Although the data from multiple sensors can be collected during the operation of the machine, it is not the case that more data contain more useful information. Some of the sensor monitoring data may not be correlated with the degradation of the machine, which not only fails to provide adequate information for the prediction of RUL but also generates data redundancy that leads to a decrease in the prediction accuracy of the model. Therefore, it is necessary to analyze the correlation between each sensor and the RUL of the machinery first and identify the sensor data with a strong correlation with the RUL. (2) The over-dimensionality and nonlinearity of data features generated by the operation of complex systems with multiple components and multiple states reduce the effectiveness of deep learning algorithms for RUL prediction. Due to the complexity of the system, the resulting data are highly dimensional and nonlinearly correlated. In feature extraction and data processing, high-dimensional feature vectors often lead to dimensional disasters [23]. As the dimensionality of the dataset increases, the number of samples required for algorithm learning increases exponentially. In addition, the sparsity of the data increases as the dimensionality increases. It is more challenging to explore the same dataset in a high-dimensional vector space than in an equally sparse dataset. Therefore, reducing the dimensionality of the data and extracting their main feature components are urgent problems.

In order to solve the limitations of the above problem, this paper explores the life prediction based on the deep long short memory (DLSTM) network with the example of aero-engine life prediction. The rest of this paper is organized as follows. Section 2 summarizes the related work and briefly introduces the proposed framework of MKDN. Section 3 details the theory of MKDN. Section 4 shows the experimental results of the proposed method by using the public Commercial Modular Aero-Propulsion System Simulation (C-MAPSS) datasets. Section 5 provides a detailed comparison and analysis of the proposed methods. Finally, conclusions and future works are drawn in Section 6.

## 2. The Related Work and Proposed Framework

To overcome the above shortcomings, a general three-step solution, MIC-KPCA-DLSTM-based neural network (MKDN), is proposed in this paper. In the first step, considering the correlation impact between features and RUL information, the maximum information coefficient (MIC) method is applied to select the key features. Then, kernel principal component analysis (KPCA) is applied for nonlinear feature extraction and dimensionality reduction. By reducing the dimensionality, over-fitting caused by excessive model parameters can be effectively eliminated. Finally, we propose a DLSTM-based deep learning method to predict RUL as the third step by inputting the above dimensionality-reduced feature data. To validate the proposed MKDN model, we conducted a case study, i.e., RUL prediction for turbofan engines. The experimental results of RUL prediction show that the MKDN model achieves a high RUL prediction accuracy and outperforms some state-of-the-art RUL prediction methods and typical deep learning models. The main contributions of this paper are summarized as follows.

(1) A robust RUL prediction framework, MKDN, is proposed by utilizing effective data processing techniques and dynamic deep learning models for time series analysis such as MIC, KPCA, and DLSTM neural network. Meanwhile, in the MKDN, a two-layer DLSTM-based prognostic model (TDPM) was designed and utilized for performance degradation and remaining useful life prediction of machinery. The proposed MKDN framework produces better results compared with state-of-the-art methods.

(2) For selecting the RUL correlation features of machinery with non-linear correlation data from multiple sensors, a two-step feature selection approach based on the maximum information coefficient theory (TFMIC) is proposed. The first step constructs a threshold function based on the MIC method. In the second step, the original data of time series variables collected by mechanical sensors are filtered by the above threshold function, and the obtained time series variables with a high impact on mechanical RUL are composed into a new feature set. Benefitting from the powerful selection capability of MIC for non-linear correlation data, the introduction of MIC in the TFMIC method not only effectively selects different sensor data that have a solid intrinsic relationship with RUL but also dramatically reduces the number of the features. Consequently, the updated feature set avoids elements with minimal significance to RUL and substantially increases the precision of RUL prediction.

(3) A coupled method based on KPCA with SMA (SKPCA) is applied to noise reduction, and feature extraction of multi-sensor data is proposed. In the method, a simple moving average method (SMA) with a sliding window is used for noise reduction and smoothing of multidimensional sensor data with large random fluctuations and noise perturbations. Then, the KPCA is applied as the second step for nonlinear feature extraction and dimensionality reduction. By extracting features and noise reduction, factors containing more overlapping information can be effectively eliminated to increase the stability of training data and also help reduce the dimensionality of training data to prevent overfitting in the training process and improve the prediction accuracy.

**Figure 1:**
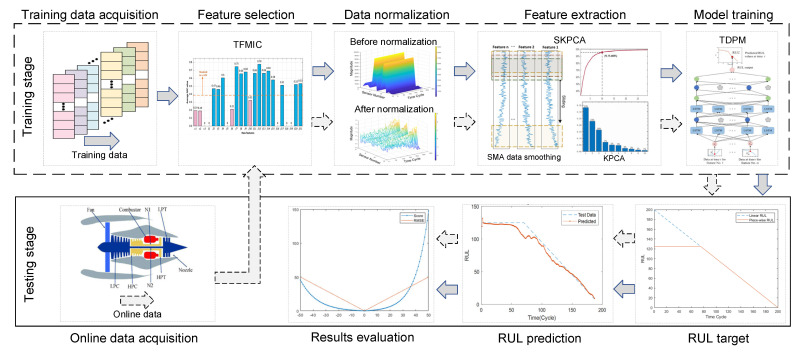
The framework of the proposed MKDN method.

As shown in Figure 1, the proposed RUL prediction framework based on MKDN for the mechanical system mainly includes data acquisition, feature selection, data normalization, noise reduction and feature extraction, model training, and RUL prediction.

During the operation of machinery, various kinds of signal data are collected by various types of sensors, such as pressure, temperature, vibration, speed, flow, and static electricity. Firstly, the TFMIC method is initially used to analyze the correlation between the collected individual sensor signals and the RUL of the machine. Then, the sensor signals strongly correlated with the RUL information are selected as the following input features. Secondly, the selected features are put into the SKPCA method to reduce noise, extract valuable features, and reduce data dimensionality. Thirdly, these extracted and dimensioned features are fed into the TDPM, which contains two LSTM layers to catch temporal features. Two fully connected layers and a regression layer are employed as the output layer, where the temporal output features are sent into them, and the gathered individual sensor signals are eventually fused to the RUL values. During the final testing phase, online sensor signal data are sequentially transmitted into the trained MKDN, and the estimated RUL is obtained. To avoid overfitting when training MKDN, a regularization method, dropout [24], is used.

## 3. The Proposed MKDN

### 3.1. Problem Formulation

During manufacturing, machinery is impacted by internal elements and the external environment, resulting in reduced performance. The performance indicators will be reduced accordingly until their eventually fails and loses its ability to work ultimately. These machines’ field operation monitoring data, which can be thought of as a time series based on the operation of the equipment, will be collected during the actual operation process by various sensors.

Given a group of machines, the monitoring dataset *S* can be described by the following equations:

(1) $$S=\left\{N_{1},N_{2},\cdots ,N_{i},\cdots ,N_{n}\right\},i=(1,2,\cdots ,n)$$

(2) $$N_{i}=\left\{X_{i1},X_{i2},\cdots ,X_{ij},\cdots ,X_{im}\right\},j=(1,2,\cdots ,m)$$

(3) $$X_{ij}={\left[x_{ij}^{1},\cdots ,x_{ij}^{k},\cdots x_{ij}^{t}\right]}^{T}(k=1,2,\cdots ,t)$$

where *N_i_* denotes the monitoring data of the *i*th machine, *n* denotes the number of machines in the monitoring data, *m* denotes the number of condition monitoring variables of each machine, *X_ij_* is the time series of the *j*th condition monitoring variable of the *i*th machine, *t* denotes the number of data samples arranged by time series, and *x_ij_^k^* is the detection value of the *j*th monitoring variable of the *i*th monitoring machine at the *k*th moment.

On the basis of the above settings, the remaining life set Y corresponding to the monitored mechanical condition data can be expressed in Equations (4) and (5) as

(4) $$Y=\left\{Y_{1},Y_{2},\cdots ,Y_{i},\cdots ,Y_{n}\right\},i=(1,2,\cdots ,n)$$

(5) $$Y_{i}={\left[y_{i}^{1},\cdots ,y_{i}^{k},\cdots y_{i}^{t}\right]}^{T}(k=1,2,\cdots ,t)$$

where *Y_i_* denotes the RUL of the *i*th machine, *y_i_^k^* is the lifetime value of the *i*th machine at moment *k*, *n* denotes the number of machines in the monitoring data, and *t* denotes the number of data samples corresponding to one time series.

### 3.2. Feature Selection Method Based on MIC

MIC is a mutual-information-based measure of the correlation between two-dimensional variables proposed by David N. Reshef [25] in 2011. Compared with traditional correlation measures such as Pearson and Spearman correlation coefficients, the MIC algorithm can not only measure linear or nonlinear relationships between variables in a large amount of data but also extensively explore the nonfunctional dependencies between variables so that MIC can measure variables with complex correlations more accurately.

#### 3.2.1. MIC Theory

MIC is calculated using mutual information and grid partitioning. Mutual information can be considered as the amount of information contained in one variable about another variable. The calculation equations of the MIC can be expressed as follows.

(1) Mutual Information

Given the variables $X=\{x_{i},i=1,2,\ldots n\}$ and $Y=\{y_{i},i=1,2,\ldots ,n\}$, where *n* is the number of samples, its mutual information $I\left(X,Y\right)$ is calculated as shown in Equation (6).

(6) $$I(X,Y)=\sum_{y\in Y}\sum_{x\in X}p(x,y)\log\frac{p(x,y)}{p(x)p(y)}$$

where $p\left(x,y\right)$ is the joint probability density of *X* and *Y*, and $p\left(x\right)$ and $p\left(y\right)$ are the marginal probability densities of *X* and *Y*, respectively.

(2) Calculation of MIC

Given a finite ordered set $D=\left\{\left(x_{i},y_{i}\right),i=1,2,\cdots ,n\right\}$, the scatter plot composed of $x_{i}$ and $y_{i}$ is x*y gridded to obtain the grid *G*. Calculate the mutual information *I(X, Y)* of each grid based on the grid *G*, and obtain the maximum mutual information $max\;I\left({\left.D\right|}_{G}\right)$, denoted as $I^{*}\left(D,x,y\right)$. The maximum mutual information on different size grids *G* is then normalized to obtain the feature matrix $M{\left(D\right)}_{x,y}$ of the two-dimensional dataset *D*:

(7) $$M{\left(D\right)}_{x,y}=\frac{I^{*}(D,x,y)}{\log\min\{x,y\}}$$

The largest one from $M{\left(D\right)}_{x,y}$, which is the maximum information coefficient $\left(D\right)$:

(8) $$MIC(D)=\underset{x*y<B(n)}{\max}\left\{M{\left(D\right)}_{x,y_{}}\right\}$$

According to Reshef [25], the general case can be taken as $B\left(n\right)=n^{0.6}\;$.

#### 3.2.2. The Proposed TFMIC

Since, in the complex failure mode, specific characteristics may be strongly associated with one another but weakly correlated with the RUL, this indicates that these features are invalid and may not perform well when estimating the RUL. In order to obtain the most representative set of features with RUL, the collected raw data need to be selected. The TFMIC method selects raw data through the following two steps: Firstly, a threshold function is constructed on the basis of the MIC method. Secondly, the original data are filtered by the threshold and composed into a new feature set.

(1) The threshold function constructed on the basis of the MIC method is divided into three main steps. The first step is to calculate the MIC values $\sigma_{ij}$ for each time series variable and RUL for each machine in the dataset. The second step is to calculate the average of the MIC values $M_{j}$ of all mechanical time series variables with RUL. The third step is to calculate the average of all $M_{j}$ to obtain the threshold $\sigma_{mic}$. The details are as follows.

(a) Calculate the MIC values $\sigma_{ij}$ for each time series variable and RUL for each machine in the dataset and obtain the MIC value matrix *D*. From Equations (3), (5) and (8), we can obtain Equation (9):

(9) $$\sigma_{ij}=MIC(X_{ij},Y_{i})$$

where *MIC*(*X_ij_,Y_i_*) denotes the MIC calculation for a time series variable *X_ij_* and remaining useful life *Y_i_*, and $\sigma_{ij}$ denotes the value of *X_ij_* and *Y_i_* after MIC calculation.

From Equations (1), (4) and (9), we can obtain the MIC value matrix *D*:

(10) $$D=MIC(S,Y)=\left[\begin{array}{cccccc} \sigma_{11} & \sigma_{12} & \cdots & \sigma_{1j} & \cdots & \sigma_{1m}\\ \sigma_{21} & \sigma_{22} & \cdots & \sigma_{2j} & \cdots & \sigma_{2m}\\ \vdots & \vdots & & \vdots & & \vdots \\ \sigma_{i1} & \sigma_{i2} & \cdots & \sigma_{ij} & \cdots & \sigma_{im}\\ \vdots & \vdots & & \vdots & & \vdots \\ \sigma_{n1} & \sigma_{n2} & \cdots & \sigma_{nj} & \cdots & \sigma_{nm} \end{array}\right]$$

(b) Calculate the average of MIC values $M_{j}$ for all mechanical time series variables with RUL. Let $M_{j}$ be the mean value calculated for all elements within $D_{j}$. From Equations (11) and (12), we obtain Equation (13):

(11) $$D_{j}=\left[\begin{array}{c} \sigma_{1j}\\ \sigma_{2j}\\ \vdots \\ \sigma_{ij}\\ \vdots \\ \sigma_{nj} \end{array}\right]$$

(12) $$D=\left[\begin{array}{cccccc} D_{1} & D_{2} & \cdots & D_{j} & \cdots & D_{m} \end{array}\right]$$

(13) $$M_{j}=\frac{1}{n}\times \sum_{i=1}^{n}\sigma_{ij}$$

(c) Calculate the threshold value $\sigma_{mic}$:

(14) $$\sigma_{mic}=\frac{1}{m}\times \sum_{j=1}^{m}M_{j}$$

(2) The $\sigma_{mic}$ calculated by Equation (14) is used as the threshold value for feature selection. If the time series characteristics obtained by each sensor fulfill Equation (15), they will be selected for addition to the new dataset *S_new_*.

(15) $$MIC(X_{ij},Y_{i})\geq \sigma_{mic}$$

### 3.3. Noise Reduction and Feature Extraction

According to the introduction of the research, the raw data are typically characterized by high dimensionality, nonlinearity, and excessive noise, which makes accurate prediction challenging. In order to solve the problem mentioned above, this study presents a coupling approach based on SMA and KPCA (SKPCA) for data processing to reduce noise and extract the data’s most important information. The SKPCA approach focuses primarily on data smoothing by SMA to reduce noise, followed by feature extraction and dimensionality reduction of high-dimensional and nonlinear data by KPCA.

#### 3.3.1. Sensor Data Smoothing

Large random fluctuations and noise disturbances in the machine’s multi-sensor data might impact the performance of RUL prediction. A time-sliding window [26] can be used to smooth data in a way that gets rid of noise and reduces fluctuations. As a window of a certain length moves through the input signal over time, it captures information about a particular instance and feeds it into the model to predict the corresponding RUL.

#### 3.3.2. KPCA

High-dimensional feature data collected by multiple sensors are prone to a nonlinear correlation between features. In order to reduce feature information redundancy and improve feature differentiation, we can try to reduce the dimensionality of the data before building the model. Being a generalization of the principal component analysis (PCA) [27] method, the KPCA [28] method is a better choice for principal element extraction for features with a high degree of nonlinearity.

The KPCA method introduces a nonlinear mapping of kernel functions to map the original features to a high-dimensional space F. The conversion from low-dimensional linearly inseparable to high-dimensional linearly separable is followed by a linear dimensionality reduction using the PCA method. Therefore, the KPCA method can effectively preserve the original data features and extract the nonlinear relationships embedded in the original features.

Given a sample set $X=\left\{x_{1},x_{2},\ldots ,x_{m}\right\}$ and a new coordinate system after transformation is $\left\{w_{1},w_{2},\ldots ,w_{m}\right\}$, where $w_{i}$ is the standard orthogonal basis vector, the projection of $x_{i}$ in the new space is $W^{T}x^{i}$, and the variance of the projected sample points $x_{i}$ can be expressed in Equation (16) as

(16) $$\sum_{i}^{n}\left(W^{T}x^{i}x_{i}^{T}W\right)$$

Applying the Lagrange multiplier method yields Equation (17):

(17) $$XX^{T}W=\lambda W$$

Performing eigenvalue decomposition on the covariance matrix $XX^{T}$ yields eigenvalues $\lambda_{1}\geq \lambda_{1}\geq \ldots \lambda_{m}$. The variance contribution rate and cumulative variance contribution rate both determine the number of principal components selected and are expressed mathematically in Equations (18) and (19), respectively.

(18) $$\eta_{i}=\frac{100\%\lambda_{i}}{\sum_{m}\lambda_{i}}$$

(19) $$\eta_{T}=\sum_{i=1}^{p}\eta_{i}$$

where $\eta_{i}$ denotes the contribution rate of the *i*th principal element in the feature set, and $\eta_{T}$ denotes the cumulative contribution of the first *p* principal elements.

#### 3.3.3. The Proposed SKPCA

As mentioned earlier, the raw data collected during the operation of machinery has high noise and high-dimensional nonlinear characteristics that can negatively impact mechanical RUL prediction, so it requires smoothing noise reduction and fusion dimensionality reduction of the data.

SMA is a smoothing method that effectively reduces the collected data’s noise. It is an operation that operates on a time-series average, including several quantities in a sequential manner during the continuous evolution of the time series and predicts the long-term trend. In the actual time series data, irregular fluctuations often occur and can significantly impact the prediction results. SMA [29] can reduce and avoid the influence of erratic changes, thus ensuring the accuracy of the forecasts of the long-term trends of the time series. Section 4.6 graphically shows the noise reduction effect of SMA.

The high-dimensional features obtained from feature engineering are prone to the linear correlation between features. To reduce feature information redundancy and improve feature differentiation before building the model, we can try to reduce the dimensionality of the data. Compared with other data dimensionality reduction and feature extraction methods such as PCA, the KPCA method is a better choice to extract nonlinear feature primitives containing the primary data information by effectively eliminating the redundancy and spatial correlation between the data.

Given that SMA can effectively perform noise reduction on mechanical multidimensional degradation data and KPCA can perform information fusion and dimensionality reduction on multidimensional automatic monitoring data, the combination of SMA and KPCA can effectively improve the prediction accuracy of RUL. To conduct noise removal, feature extraction, and dimensionality reduction from raw data, we proposed an SKPCA method in this work.

The proposed SKPCA method is implemented in two parts. Initially, the data undergo noise reduction using Equation (20). Then, the KPCA approach is used to perform feature extraction and data dimensionality reduction on the noise-reduced data.

In the SKPCA approach, the data are processed within each time sliding window using the simple moving average (SMA) method [29], a typical data smoothing tool for analyzing time series in technical analysis. The formula is outlined as follows:

(20) $$SMA_{t}=\frac{1}{n_{sw}}\sum_{i=t-n_{sw}+1}^{t}x_{i}$$

where *t* represents moment *t* in the time series, *n_sw_* represents the sliding window size, $x_{i}\;$ represents the actual acquisition value at the moment *i*, and $SMA_{t}$ represents the moving average at moment *t.*

**Figure 2:**
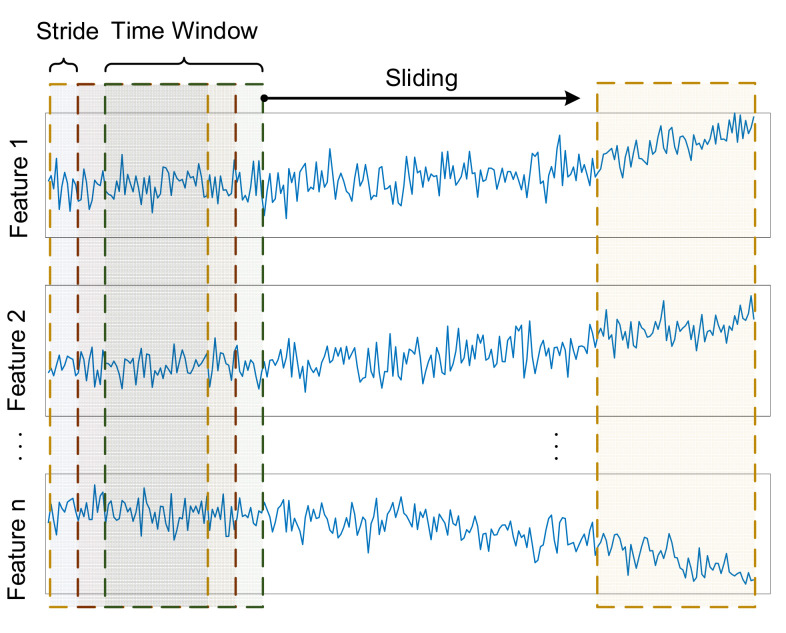
The processing of the time sliding window.

The processing of the time sliding window is shown in Figure 2, where the window size *n_sw_* slides along the time series, and SMA method smooths the data inside the window for each sliding step. These smoothed data will be used as the input data for the subsequent steps of the model. The step length of the window is referred to as stride in this work.

Following that, KPCA was used to process the smoothed data. For our research, we adopted the well-known Gaussian (RBF) kernel [28], which has highly robust representational capabilities. The formula is outlined as follows:

(21) $$K(x,y)=\exp\left(-\frac{∥x-y{∥}^{2}}{2\sigma^{2}}\right)$$

By calculating the cumulative contribution of the features, an acceptable threshold $\sigma_{kpca}$ is determined for selecting the desirable features using Equation (22). All of the features calculated by KPCA are ranked in descending order of the contribution rate of each feature, and when the first *q* features’ cumulative contribution rate $\eta_{T}$ reaches this threshold $\sigma_{kpca}$, the first *q* features are formed into a new optimal feature set.

(22) $$\eta_{T}\geq \sigma_{kpca}$$

The newly constructed optimal feature set can be used as an input feature vector for the designed DLSTM prognostics model.

### 3.4. The DLSTM Prognostic Model

#### 3.4.1. LSTM

LSTM [14] is a specific RNN architecture designed to model time series and their long-range dependencies more accurately than traditional RNNs. In the LSTM network structure, the LSTM unit constructs the LSTM layer instead of the traditional RNN hidden neurons, and each LSTM neuron has three well-designed gate functions, namely, input gate, forgetting gate, and output gate. This structure guarantees that the LSTM unit can discover and remember long-term interdependencies.

**Figure 3:**
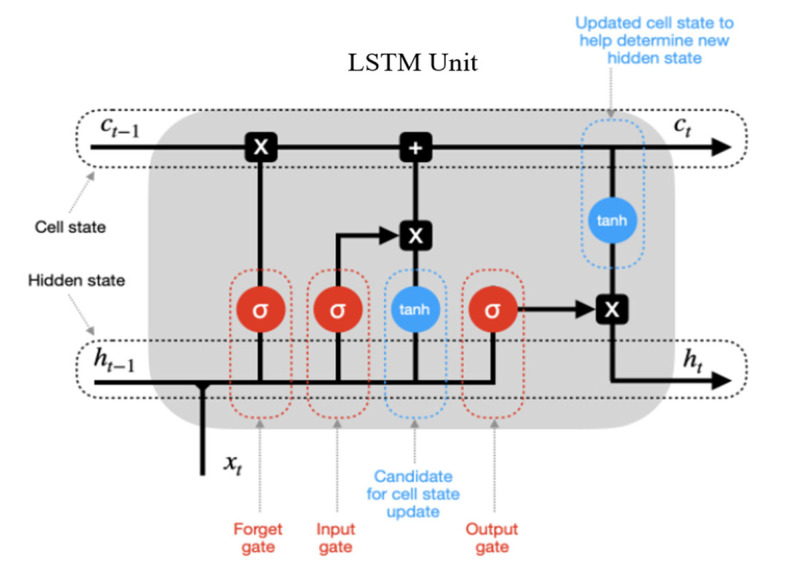
The structure of the LSTM unit.

Figure 3 illustrates the structure of the LSTM unit. With the input gate that parses the information input to the LSTM neuron, the forgetting gate that determines which information in the neuron needs to be dropped, and the output gate that determines which information is output, the three gate functions in the LSTM unit provide a suitable nonlinear regulatory mechanism for controlling the information input and output. Equations (23)–(28) present the mathematical computation process in the LSTM network.

(23) $$g_{t}=\varphi \left(w_{gx}x_{t}+w_{gh}h_{t-1}+b_{g}\right)$$

(24) $$i_{t}=\sigma \left(w_{ix}x_{t}+w_{ih}h_{t-1}+b_{i}\right)$$

(25) $$f_{t}=\sigma \left(w_{fx}x_{t}+w_{fh}h_{t-1}+b_{f}\right)$$

(26) $$o_{t}=\sigma \left(w_{ox}x_{t}+w_{oh}h_{t-1}+b_{o}\right)$$

(27) $$c_{t}=g_{t}⊙i_{t}+c_{t-1}⊙f$$

(28) $$h_{t}=\varphi \left(c_{t}\right)⊙o_{t}$$

where $w_{gx},w_{fx},w_{ix}$, and $w_{gx}$ are weights of input data $x_{t};w_{gh}$, $w_{fh},w_{ih}$, and $w_{oh}$ are weights of the previous output $h_{t-1}$ of LSTM unit; $b_{g},b_{f},b_{i}$, and $b_{0}$ indicate the bias of input node, forget gate, input gate, and output gate, respectively; $g_{t},f_{t},i_{t}$, and $o_{t}$ are the output of input node, forget gate, input gate, and output gate, respectively; σ and φ represent the sigmoid and tanh function, respectively; $c_{t}$ and $c_{t-1}$ are the LSTM neuron states at time t and t−1, respectively; and ⊙ represents the pointwise multiplication.

#### 3.4.2. DLSTM

**Figure 4:**
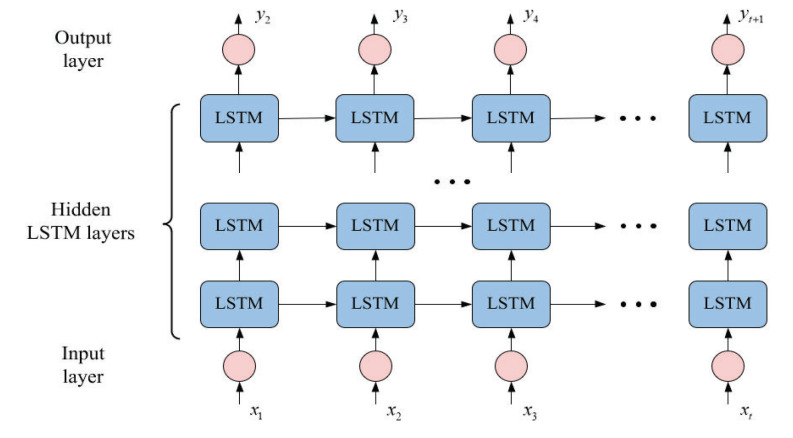
The structure of the DLSTM network.

In recent years, DLSTM has been constructed by stacking multiple LSTM layers, and this deep architecture has been proven to be successful in representation learning [30]. The core idea behind deep neural networks is that the input to the model should pass through multiple nonlinear layers so that the input to a deep LSTM model can pass through multiple LSTM layers. As shown in Figure 4, the output of each layer is transmitted to the neighboring LSTM unit and the layer directly above it. The hidden output of one LSTM layer is not only propagated through time but also used as input data for the next LSTM layer. LSTM layer stacking has two advantages. One is that the stacked layers enable the model to learn the features of the original signal on different time scales. The other is that the parameters can be distributed spatially, i.e., upon the layers, without increasing the memory size, which helps perform more efficient nonlinear operations on the raw input signal.

#### 3.4.3. The Proposed TDPM

**Figure 5:**
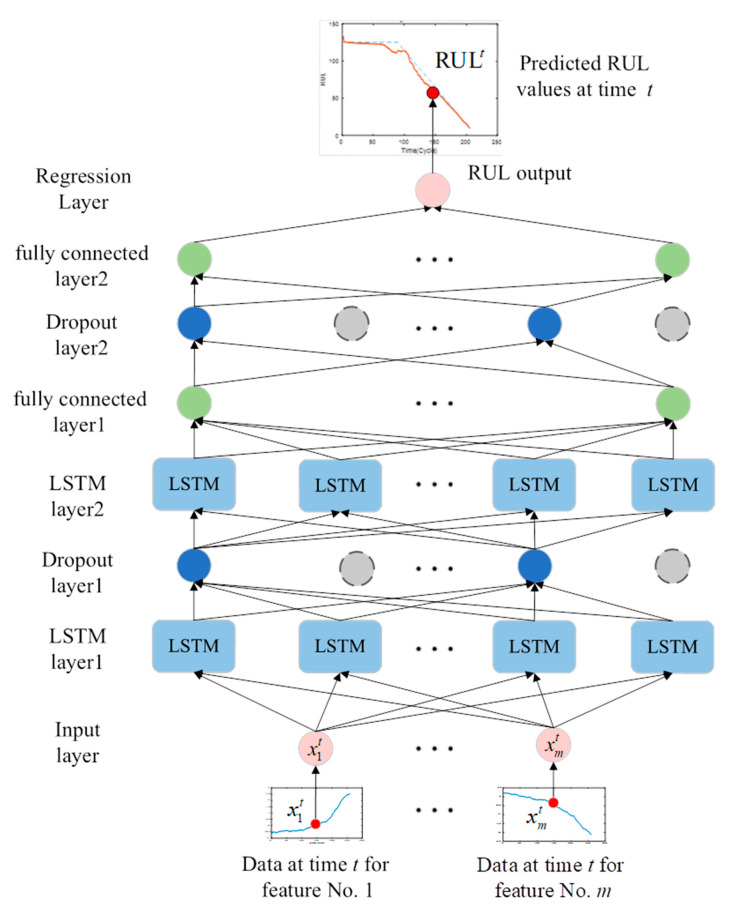
Illustration of the TDPM prognostic model.

In this study, a two-layer DLSTM-based prognostic model (TDPM) was constructed to evaluate performance degradation and predict the RUL of the engine with multiple sensors. The TDPM is employed as the fundamental prediction model in the proposed MKDN. It can successfully simulate the nonlinearity of the input data and consists of three components. The first part is two LSTM layers, which are used to learn the long-term dependencies from the data output of SKPCA. The second part is two fully connected layers, which map the learned feature representation to the label. The third part is a regression layer, which evaluates performance degradation and predicts the actual RUL. The structure of the two-layer DLSTM-based prognostic model is shown in Figure 5. In order to overcome the overfitting problem between LSTM layers and fully connected layers, the dropout method [24] was applied to the TDPM to prevent the capture of the same features repeatedly.

### 3.5. MKDN Training Process

As with conventional neural network training for regression tasks, we employed the mean squared error (MSE) loss function [31] in our MKDN architecture to determine the optimal parameters:

(29) $$\mathrm{MSE}=\frac{1}{n}\sum_{i=1}^{n}d_{i}^{2}$$

where n is the number of training samples, and $d_{i}={\hat{t}}_{iRUL}-t_{iRUL}$ is the error between the estimated RUL and the actual RUL with respect to the *i*th testing sample.

We employed the mini-batch gradient descent method [31] and the Adam algorithm [32] for optimization purposes. In the training process, the input dataset is separated into a training set for training the model and a validation set for evaluating the model’s performance. Last but not least, the hyperparameters with the most satisfactory validated prediction performance are employed for online RUL prediction.

## 4. Experiment Analysis

### 4.1. Dataset Description

**Figure 6:**
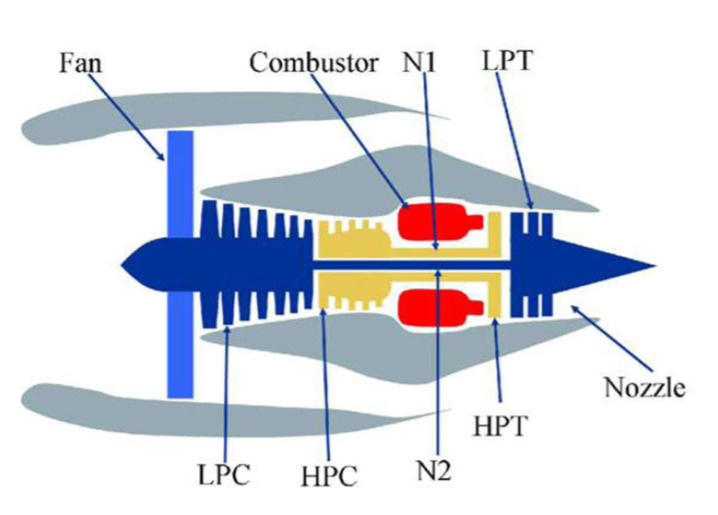
Diagram of gas turbofan engine modules.

NASA turbojet datasets generated by the commercial modular aero-propulsion system simulation (C-MAPSS) platform [33] were utilized to evaluate the proposed method. It is one of the most widely utilized forms of benchmark data in RUL prediction studies. As seen in Figure 6, the C-MAPSS platform was simulated using a typical gas turbofan engine consisting of five modules: fan, low-pressure compressor (LPC), high-pressure compressor (HPC), low-pressure turbine (LPT), and high-pressure turbine (HPT). Different operational parameters, such as fuel velocity and pressure, are varied to model various failure and degradation processes in turbofan engines. During the experiment, the turbofan engine begins running in good condition and gradually develops anomalous states that lead to deterioration and eventual failure.

The datasets were divided into four subsets, numbered FD001 through FD004, each with its training and test subsets, as indicated in Table 1. The training datasets contained the signal for the entire lifetime, while the test datasets contained the entire sensor data ended at some point before the engine failure, wherein the RUL needed to be predicted. The training and test datasets consisted of several cycles, each containing 26 columns representing the engine’s ID, cycle index, 3 operating parameters, and 21 sensor measurements.

### 4.2. Performance Evaluation Indicators

To evaluate the performance of the proposed MKDN model for RUL prediction and to facilitate comparison with other methods, two commonly used evaluation criteria, namely, the root mean square error (RMSE) and the scoring function, were applied and introduced as follows.

(1) Scoring function: The scoring function utilized in this work is defined in the 2008 Prognostics and Health Management Data Challenge [33], which is expressed as
  (30) $$Score=\left\{\begin{array}{l} \overset{n}{\underset{i=1}{\sum }}e^{-\left(\frac{d_{i}}{13}\right)}-1\;\mathrm{for}\;d_{i}<0\\ \overset{n}{\underset{i=1}{\sum }}e^{\left(\frac{d_{i}}{10}\right)}-1\;\mathrm{for}\;d_{i}\geq 0 \end{array}\right\}$$
  where *score* is the computed value of scoring function, *n* is the number of testing samples, and $d_{i}={\hat{t}}_{iRUL}-t_{iRUL}$ is the error between the estimated RUL and the actual RUL concerning the *i*th testing sample.
(2) RMSE: The *RMSE* is a widely used metric for performance assessment in prognostics and health management. The *RMSE* can be measured as follows:
  (31) $$RMSE=\sqrt{\frac{1}{n}\sum_{i=1}^{n}d_{i}^{2}}$$

### 4.3. RUL Target Function

**Figure 7:**
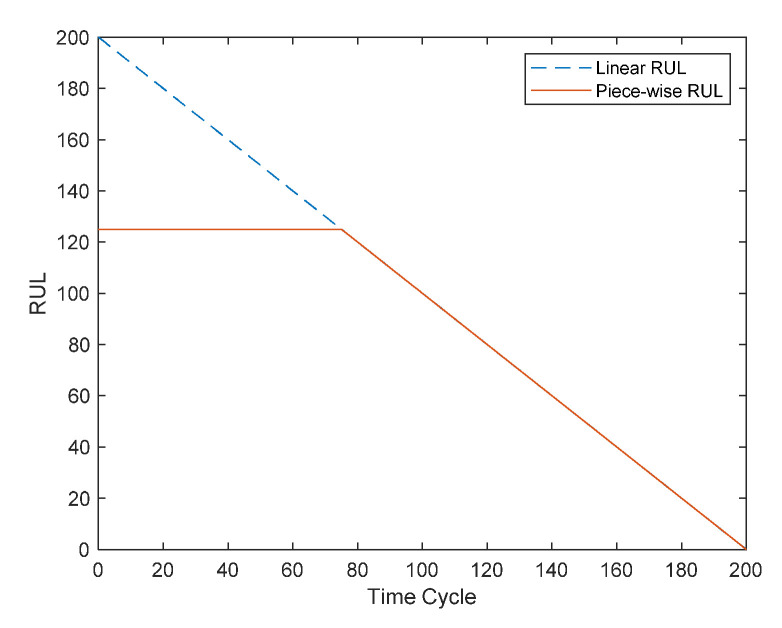
Piecewise linear model.

The segmented linear model is used for prediction [31], as shown in Figure 7. The segmented linear model is utilized because the engine’s deterioration characteristics are not immediately apparent at first. Instead, after a period of time, the engine’s level of degradation typically worsens until failure.

### 4.4. The Results of Feature Selection Obtained by TFMIC

Low correlation between features and RUL in the C-MAPSS datasets may cause unsatisfactory RUL estimation performance. Therefore, features reflecting mechanical degradation should be found to obtain accurate predictions.

Initially, the feature set *S* = {degradation life cycles, condition1, condition2, condition3, sensor1, sensor2, …, sensor21} constructed by the raw data is built as the input dataset of the TFMIC algorithm. For the convenience of calculation, we ranked the degradation life cycles of each engine from largest to smallest as the RUL of this engine. The operating conditions data and sensor features are ordered on the basis of their location in *S*, namely, *c*1*, c*2*, c*3*, f*1,..., *fi*,..., *f*21*,* where *fi* denotes the *i*th feature.

The TFMIC method is developed to solve the deficiency of inadequate consideration of nonlinear interactions and to mine the deep mutual information between features and degrading life cycles. Taking the FD003 dataset in C-MAPSS datasets as an example, we denoted the training data in the FD003 dataset by *S*, where *n* denotes the number of aero engines, *m* denotes the number of condition monitoring variables for each engine, *N_i_* denotes the monitoring data of the *i*th engine, and *Y_i_* denotes the RUL of the *i*th engine. So, we can obtain *n* = 100, *m* = 24, *S*= {*N_1_*, *N_2_, …N_i_,…N_100_*}, *Y* = {*Y_1_, Y_2_,…, Y_i_,…Y_100_*}, *N_i_* = {*c*1, *c*2, *c*3, *f*1, …, *fi*, …, *f*21}. Then, calculating the MIC threshold values between each feature and the deterioration life cycle achieves the main feature subset, where features that vary little with the deterioration life cycle are excluded. The threshold in the TFMIC method is obtained by Equation (14) and finds $\sigma_{mic}$ = 0.39.

**Figure 8:**
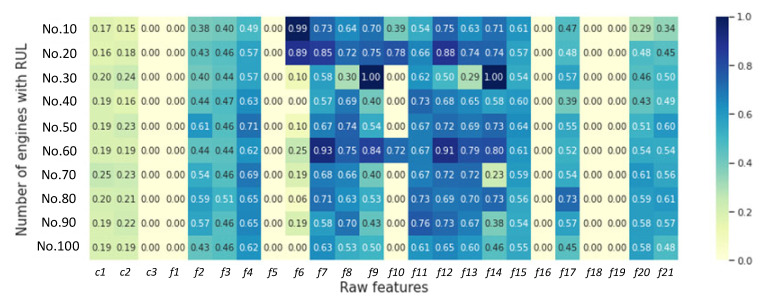
Heatmap of MIC value between features and URL for 10 engines in FD003.

**Figure 9:**
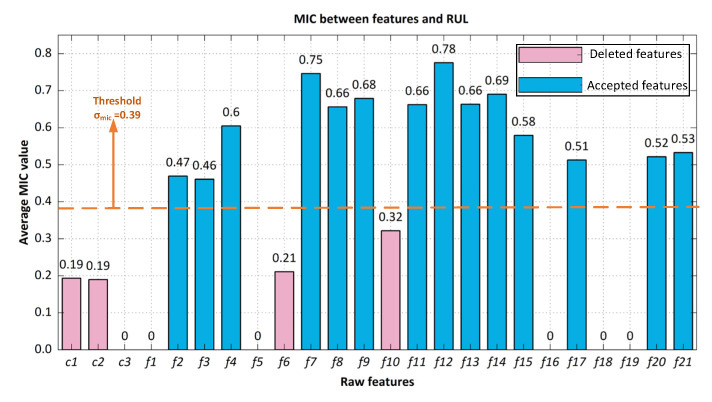
The average MIC value between each feature and life cycle.

Figure 8 shows the MIC calculation results for each feature with RUL for 10 of the engines in FD003 dataset nos. 10–100. Meanwhile, as seen in Figure 9, the threshold of the FD003 dataset was computed to be 0.39 to weed out features rarely associated with life cycles. Finally, the new optimal feature set were acquired as *S_op_* = [*f*2*, f*3*, f*4*, f*7*, f*8*, f*9*, f*11*, f*12*, f*13*, f*14*, f*15*, f*17*, f*20*, f*21].

### 4.5. Data Normalization

Since the acquired sensor data have different ranges, a normalization process is required to unify the values and obtain unbiased information from the readings of each sensor. In this study, the z-score normalization method [34] was used to obtain the standard range of all variables.

(32) $$x_{\mathrm{norm}}^{t}=\frac{x^{t}-\mu^{t}}{\sigma^{t}}$$

where $x^{t\;}$ represents the original signals collected for the *t*-th sensor; $x_{\mathrm{norm}}^{t}$ represents the standardization data; and $\mu^{t}$ and $\sigma^{t}$ denote the mean and standard deviation of $x^{t}$, respectively. Normalization helps to ensure that all variables associated with all operating conditions are considered equally.

### 4.6. The Results of Noise Reduction and Feature Extraction

**Figure 10:**
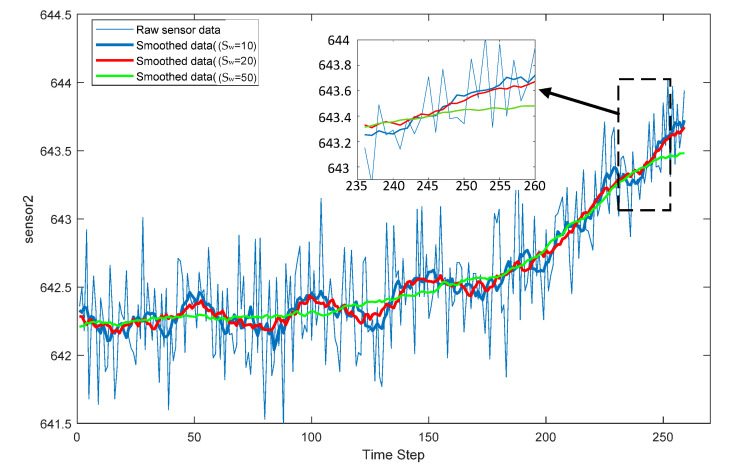
Raw sensor data and smoothed data with various S_w_ of sensor 2 in FD003.

This study used the SKPCA method to reduce noise and extract degradation information with the interference signals eliminated. The multi-sensor data obtained by the engine has large random fluctuations and noise interference that may affect the performance of the RUL predictions. Therefore, SMA combined with a moving sliding window algorithm was used to remove the noise and attenuate the random fluctuations of the sensor data. The sliding window length (S_w_) directly determines the smoothing effect of the engine sensor data and thus directly affects the accuracy of the RUL prediction. Figure 10 shows the pre-processed data for sensor 2 with different sliding window lengths compared to the original sensor data in FD003. As shown in Figure 10, the sensor data were smoothed using three different S_w_ of 10, 20, and 50. The fluctuations in the smoothed sensor data were reduced as compared to the raw sensor data, well reflecting the trend of the raw sensor data. In addition, a series of comparison experiments in Section 4.7.1 found that better prediction values were obtained when a S_w_ of 20 was used, implying that the data smoothing effect had the best effect on the prediction when the S_w_ was 20. Therefore, in this experiment, the S_w_ was set to 20, and in order to obtain more data, the step length of the sliding window was set to 1.

KPCA was used to extract the aforementioned SMA-smoothed features and perform dimensionality reduction. The model was built by using the RBF of Equation (21) as the kernel function, and the cumulative variance contribution rate was chosen as the criterion for the selection of the target dimensionality reduction, where $\sigma^{}=\sqrt{\frac{25}{2}}$ and the threshold of cumulative contribution rate of the kernel principal element $\sigma_{kpca}$ = 95%.

**Figure 11:**
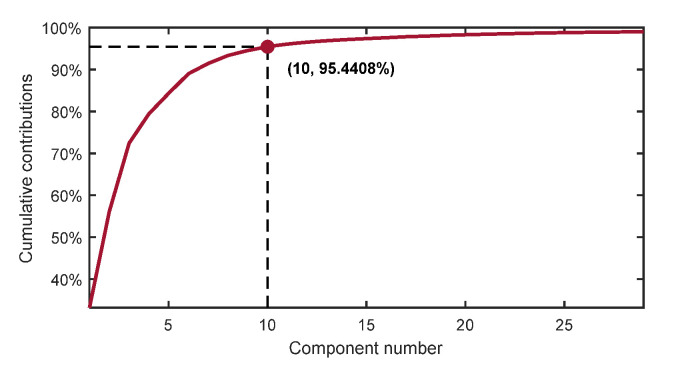
The cumulative variance contribution of the first 10-dimensional principal components.

**Figure 12:**
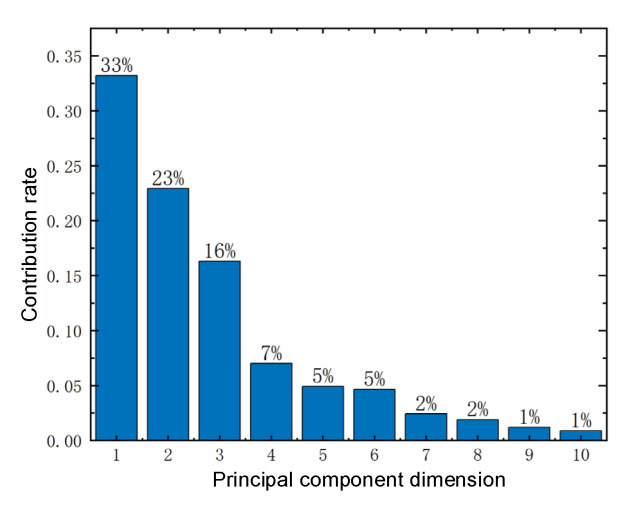
The variance contribution of each principal component in the first 10 dimensions.

The ones that satisfy the threshold condition were the first 10-dimensional kernel principal elements, and their cumulative contribution rates are shown in Figure 11. The respective contribution rates of the first 10 kernel principal elements are shown in Figure 12. Finally, these 10-dimensional data after feature extraction were selected as the optimal dataset for subsequent TDPM model training.

To reflect the superiority of SKPCA more intuitively, comparison tests were conducted using the most commonly used dimensionality reduction methods in other literature, PCA and KPCA, as well as the feature extraction methods SPCA (SMA + PCA) and SKPCA (SMA + KPCA) based on data smoothing SMA proposed in this paper. Table 2 shows the average RUL prediction errors for the 10 test engines in the FD003 dataset using the four methods mentioned above. In these experiments, the feature selection method TFMIC and prediction algorithm TDPM were used the same. Clearly, the process based on SKPCA achieved the best performance with 9.82 in RMSE and 226.55 in the scoring function. The other methods were weak in scoring function, which is unsuitable for RUL estimation as opposed to SKPCA.

**Figure 13:**
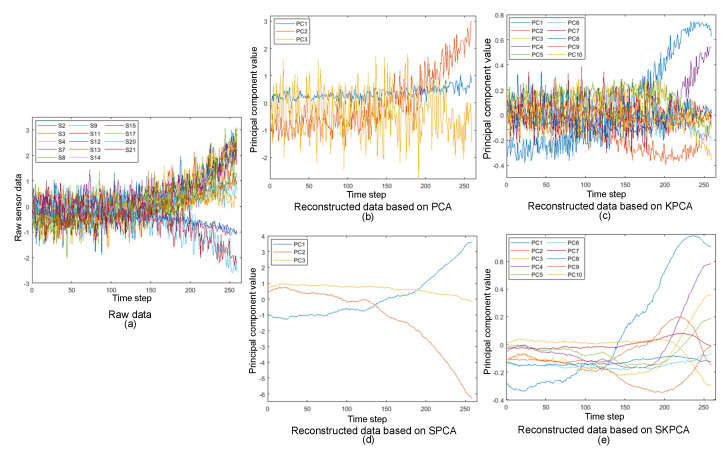
The raw and reconstructed data based on PCA, KPCA, SPCA, and SKPCA for one engine in FD003.

Figure 13 shows the raw and reconstructed data through PCA, KPCA, SPCA, and SKPCA for one engine in FD003. Figure 13a shows the raw data before the information was extracted using the four methods mentioned above, where S2–S21 represent the raw data after normalization of the 14 sensors selected by TFMIC. Figure 13b–e shows the principal component data obtained with the above four methods, where PC1-PC10 indicate the different principal component information extracted by the four methods. Figure 13b,c shows that the PCA and KPCA methods had significant fluctuations in the extracted features when SMA data smoothing was not used, which was very unfavorable for predicting the mechanical RUL. From Figure 13d,e, we can see that the features extracted by both SPCA and SKPCA were effective in noise elimination, but when the cumulative contribution of the principal element and the kernel principal element both took the same threshold of 95%, the feature information extracted by SKPCA was relatively more comprehensive than that by SPCA because there were only three principal components extracted by SPCA and 10 principal components extracted and more feature details extracted by SKPCA.

In conclusion, SKPCA can effectively reduce data fluctuation and more fully mine the data compared with other commonly used feature extraction methods so that the SKPCA algorithm can obtain more highly accurate RUL prediction results.

### 4.7. The Results of Model Prediction

The different architectures and parameters of this proposed network affect the prediction performance. Therefore, the architectures and parameters of the proposed TDPM were investigated on the C-MAPSS subset FD003. In particular, three essential factors, namely, sliding window length, batch size, and the number of LSTM layers, need to be determined.

#### 4.7.1. Effects of the Sliding Window Length

Large random fluctuations and noise disturbances in the multi-sensor data obtained from the aero-engine may affect the performance of RUL predictions. Therefore, using data smoothing methods to remove noise and attenuate the random fluctuations of sensor data is beneficial to improving prediction accuracy. According to Equation (20), combination with the time sliding window technique can effectively remove the random fluctuations and noise disturbances in the data of this example. Among them, the sliding window length (S_w_) determines the degree of data smoothing. However, the final RUL prediction obtained has a large gap using different S_w_ to smooth the data.

**Figure 14:**
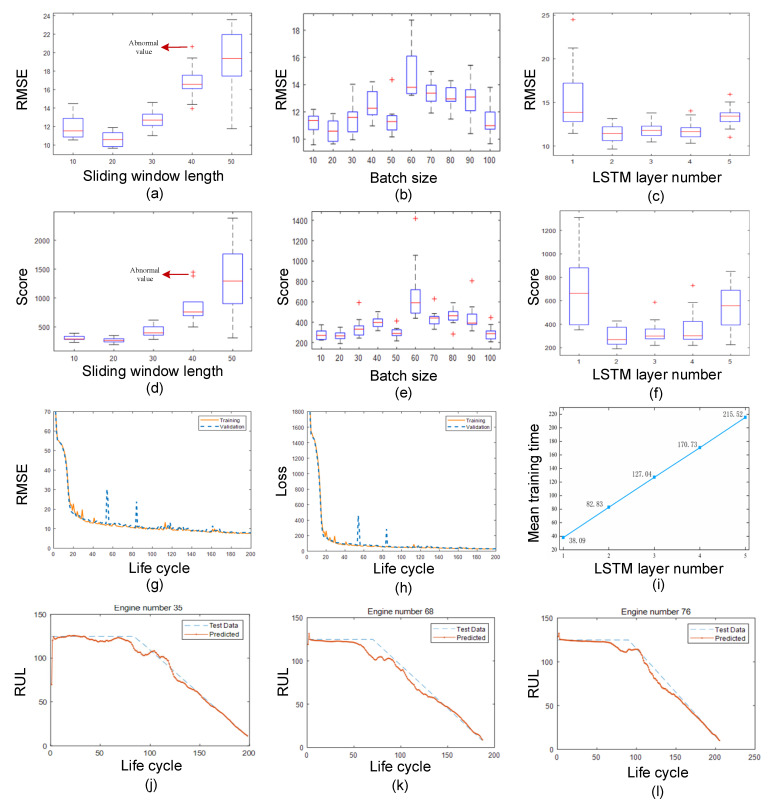
The results of evaluating RUL by TDPM with different parameters. (**a**–**c**) Box plots of RMSE values with different sliding window lengths, batch sizes, and LSTM layer numbers. (**d**–**f**) Box plots of score values in different sliding window lengths, batch sizes, and LSTM layer numbers. (**g**–**h**) The iterative process of the loss and RMSE values. (**i**) The average training time for different number of layers. (**j**–**l**) The results of RUL estimation in three random engines.

Figure 14a,d illustrates box plots of RMSE and score values for RUL estimation when the S_w_ is taken from 10 to 50. It can be seen from the plots that when the S_w_ was 20, the prediction performance was the best among the two-evaluation metrics. When it was greater than 20, the RUL prediction performance deteriorated rapidly as the S_w_ increased. This is because when SMA is used for data smoothing when the S_w_ is small, it can adequately attenuate the random fluctuations of sensor data and remove the noise well. However, as the S_w_ increased, the averaged data are so much that the data themselves become seriously distorted, leading to the rapid deterioration of RUL prediction performance. Meanwhile, when it is too small, the SMA data smoothing method is unable to effectively reduce the random fluctuations of the original sensor data and remove the noise. So, considering the two-evaluation criterion, the S_w_ was set to be 20.

#### 4.7.2. Effects of the Batch Size

Each epoch’s training duration and the degree of gradient smoothness between iterations are both determined by the batch size. Appropriate batch size parameter makes the gradient descent direction of the small batch size dataset determined by it better represent the gradient descent direction of the overall sample, thus ensuring the accuracy of the loss function in calculating the extreme value direction.

Figure 14b,e shows the box plots of RMSE and score values of RUL estimation when the batch size was taken from 10 to 100. The results show that the variation of the error did not show monotonicity with the increase in batch size. With the increase in batch size, the comprehensive performance of the prediction results showed a trend of getting worse and then better. The worst prediction was achieved when the value of batch size was 60. The all-around performance of the prediction was relatively good when the value of batch size was small, especially the performance of the score function. In addition, when the batch size was larger than 50, the probability of outliers in the score evaluation index increased, and the prediction performance became unstable. Considering the accuracy and concentration of the prediction values of RMSE and score values, its performance was the best among the two-evaluation metrics when the batch size was 20. In this study, the batch size was determined to be 20, considering the significance of the accuracy and dependability of predictive capabilities in the operation of engines.

#### 4.7.3. Effects of the LSTM Layer Number

Generally speaking, the more layers of a neural network, the deeper the abstraction level of input features and, therefore, the better the prediction effect. However, when the number of layers reaches a certain level, the prediction effect worsens due to the lack of data and overfitting. The more layers, the more resources are consumed for training and the corresponding training time is longer.

Figure 14c,f shows the box plots of RMSE and score values of RUL estimated with LSTM layers ranging from 1 to 5. The plots revealed that the RUL predictions were comparable when the LSTM layer number was 2, 3, or 4, but deteriorated when the layer number was 1 or 5. This is because the neural network cannot tap the intrinsic connection between sensor data and RUL when the layer number is too small. However, when the layer number is too large, overfitting occurs. Figure 14i shows the average training time for a different number of layers, and it is evident from the figure that the training time became longer as the layer number increased. The prediction effect was similar when the LSTM layer number was 2, 3, and 4, but the training time was shorter when the layer number was 2. In industrial applications, the shorter the computation time of the algorithm, the better it is to make decisions quickly, so the LSTM layer number was chosen to be 2.

#### 4.7.4. Final Parameter Settings and Prediction Results

By finding the optimal parameters for the proposed TDPM network architecture, the final parameter settings obtained are shown in Table 3.

Figure 14g,h shows the iterative process of the loss values and the iterative process of the RMSE values of the TDPM network architecture in the training process under this setting, where the training process contains both the training and validation sets. Figure 14j–l shows the results of RUL estimation in three random engines, where the red curve represents the predicted value and the blue curve represents the actual RUL value. It can be seen that the predicted values were distributed around the valid values when the engines were in the middle and late stages of the cycle, so the predicted values obtained by the model fitted the actual values very accurately. It can be concluded that this model has high prediction accuracy for such complex machinery as engines, which can provide a basis for improving the reliability and safety of engines.

## 5. Comparisons and Analysis

### 5.1. The Validity of Feature Construction Method TFMIC-SKPCA

In order to evaluate the efficacy of the proposed feature construction approach TFMIC-SKPCA, the prediction results obtained by the features extracted using TFMIC-SKPCA were experimentally compared with the prediction results obtained by the features employed in the existing literature. In the literature [26,35], the measurements of 14 sensors were used as input features, namely, 2, 3, 4, 7, 8, 9, 11, 12, 13, 14, 15, 17, 20, and 21, which are conventional features commonly used in the existing literature.

**Figure 15:**
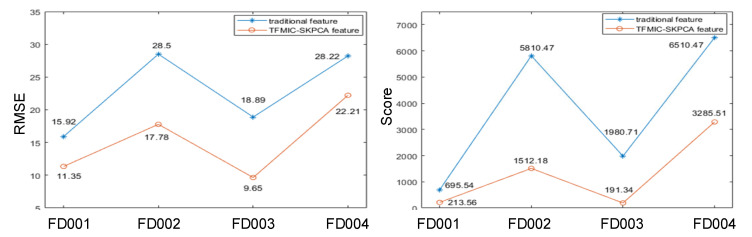
Comparison of traditional features and TFMIC-SKPCA features for RUL prediction.

Figure 15 shows the RUL prediction results yielded through the features obtained by TFMIC-SKPCA and the conventional features commonly used in the existing literature. Regarding RMSE values, the predictions based on the TFMIC-SKPCA features significantly outperformed the traditional features. For subsets FD001 and FD003, the TFMIC-SKPCA feature-based approach performed slightly better than the conventional feature-based method in terms of score values. For subsets FD002 and FD004, however, the TFMIC-SKPCA feature-based approach achieved excellent outcomes in early prediction by obtaining lower Score values under complicated operating circumstances and high noise, which is vital for the maintenance of the critical machine.

The results of the experiments demonstrate that the degraded features can be successfully recovered from the raw sensor measurement data using the proposed feature building method TFMIC-SKPCA, and sensitive features that are strongly connected with the RUL of the machinery may be chosen. According to the preceding description, the presented TFMIC-SKPCA approach offers exceptional nonlinear noise signal processing and analysis capabilities. Furthermore, it has minimal parameters and is easily adaptable to diverse datasets.

### 5.2. Comparisions with the State-of-the-Art Methods

For the purpose of establishing the validity and superiority of the proposed framework, comparisons have been made with some of the state-of-the-art methods of the past several years. The MKDN framework described in this research surpasses existing comparative approaches, achieving an RMSE of 9.65 and a score of 191.34 on the FD003 test set. The predictions of the RUL for four subsets of the C-MAPSS dataset are summarized in Table 4. Compared with the previous optimal model, the RMSE values of the MKDN model on the four datasets were reduced by 5.1%, 12.59%, 22.56%, and 0.98%, and the score values were reduced by 6.74%, 44.61%, 32.63%, and 2.53%, respectively.

Compared to SVM, MODBNE, and DCNN, which predict on the basis of local degradation features, the MKDN method proposed in this study not only has advantages in MIC feature selection and KPCA feature extraction, but it also has advantages of RNN in processing sequence information, which learns the whole degenerative trend characteristics of multi-sensor sequences.

Considering datasets with complicated failure modes, particularly FD002 and FD004, it provides a practical application with improved generalization capacity and more precise predictions. Unlike model-based approaches such as RF and GB, MKDN methods exhibit significant improvements in RMSE and score values and can adaptively uncover more complex hidden connections from sensor measurement data. DLSTM, BLSTM, and other RNN-based models perform well with low RMSE in RUL estimation; however, score performs poorly. Compared with Li-DAG, a hybrid model combining CNN and LSTM, it performs similarly in subsets FD001 and FD004. However, MKDN highlights critical information after feature selection and feature extraction and performs better overall. In conclusion, the proposed MKDN framework in this paper performs well on both evaluation metrics.

This demonstrates that the MIC-based TFMIC method can select features that are highly relevant to RUL and that SKPCA has good performance in removing noise and data fluctuations as well as extracting the primary information of the data, which leads to improved engine RUL prediction in the MKDN framework.

## 6. Conclusions

In this study, a new MKDN model based on the DLSTM network for RUL prediction of nonlinear deterioration process is proposed. In the model, TFMIC is designed to select the most relevant features, and the SKPCA is built to eliminate noise, reduce dimensionality, and extract nonlinear features. The last step is using TDPM, an optimized network with two LSTM layers and fully connected layers, to predict RUL. C-MAPSS-Data, a dataset consisting of aero-engines with a nonlinear degradation process, was utilized to evaluate the proposed method. Results show that MKDN can provide better RUL prediction for nonlinear deterioration process of complex systems. Compared with the state-of-the-art methods, the MKDN method achieves a maximum decrease in RMSE and score of 22.56% and 44.61%, respectively.

In the future, we will investigate ways to extract nonlinear information more efficiently in order to significantly increase prediction accuracy.

## Figures and Tables

**Table 1:** Details of the datasets from C-MAPSS.

Dataset	FD001	FD002	FD003	FD004
Train trajectories	100	260	100	249
Test trajectories	100	259	100	249
Conditions	1	6	1	6
Fault modes	1	1	2	2

**Table 2:** The prediction results by different methods.

	PCA	KPCA	SPCA	SKPCA
RMSE	18.33	16.17	12.83	9.82
Score	1326.95	845.61	249.79	226.55

**Table 3:** The TDPM neural network parameter settings.

Network Parameter	Value	Network Parameter	Value
Sliding window size	20	Dropout-2	0.5
Input layer units	10	Fully connected layer-2 units	100
LSTM layer-1 units	600	Regression layer unit	1
Dropout-1	0.2	Batch size	20
LSTM layer-2 units	400	Learning rate	0.001
Fully connected layer-1 units	300	Epochs	200

**Table 4:** Comparisons between different methods.

Methods	FD001		FD002		FD003		FD004	
	RMSE	Score	RMSE	Score	RMSE	Score	RMSE	Score
RF [36]	17.91	479.95	29.59	70,456.86	20.27	711.13	31.12	6567.63
SVM [36]	40.72	7703.33	52.99	316,483.31	46.32	22,541.58	59.96	41,122.19
GB [36]	15.67	474.01	29.09	87,280.06	16.84	576.72	29.01	7817.92
DLSTM [37]	16.14	338	24.49	4450	16.18	**284**	23.31	12,466
BLSTM [38]	13.65	295	**23.18**	4130	13.74	317	24.86	54,300
RNN [26]	13.44	339	24.03	14,300	13.36	347	24.02	14,300
DCNN [26]	12.61	273.7	22.36	10,412	12.64	284.1	23.31	12,466
MODBNE [36]	15.04	334.23	25.05	5585.34	12.51	421.91	28.66	6557.62
Li-DAG [39]	**11.96**	**229**	20.34	**2730**	**12.46**	535	**22.43**	**3370**
MKDN	**11** **.35**	**21** **3.56**	**17** **.78**	**1512.18**	**9.65**	**191.34**	**22.21**	**3285.51**

## Data Availability

The data presented in this study are openly available in NASA Ames Prognostics Data Repository at http://ti.arc.nasa.gov/project/prognostic-data-repository; (accessed on 10 November 2021).
